# Telemedicine in Arab Countries: Innovation, Research Trends, and Way Forward

**DOI:** 10.3389/fdgth.2020.610837

**Published:** 2021-01-25

**Authors:** Ahmed Waqas, Shariq Mehmood, Arwah Muhammad Jawwad, Bradley Pittam, Shantanu Kundu, Jorge César Correia, Nouf AlMughamis

**Affiliations:** ^1^Institute of Population Health, University of Liverpool, Liverpool, United Kingdom; ^2^Lahore General Hospital, Lahore, Pakistan; ^3^School of Electrical Engineering and Computer Sciences, National University of Science and Technology, Islamabad, Pakistan; ^4^Manchester University NHS Foundation Trust, Manchester, United Kingdom; ^5^University of Liverpool School of Medicine, Liverpool, United Kingdom; ^6^Geneva University Hospitals, University of Geneva, Geneva, Switzerland; ^7^Ministry of Health, Kuwait City, Kuwait

**Keywords:** telemedicine, digital health, Middle East, research policy, scientometric, bibliometric

## Abstract

**Background:** The progress and innovation in telemedicine within the Middle Eastern countries have not been heavily monitored. Therefore, the present study aims to analyze the scholarly work conducted in the Arab world, using reproducible statistical and scientometric techniques.

**Methods:** An electronic search of Web of Science (core database) had been conducted through use of an extensive search strategy comprising of keywords specific to the Arab region, EMRO countries, telehealth, medical conditions, and disorders. A total yield of 1,630 search results were processed, indexed through July 7, 2020. CiteSpace (5.7.R1, Drexel University, Pennsylvania, USA) is a Java-based application, a user-friendly tool for conducting scientometric analyses.

**Results:** The present analyses found a lack of innovation in the field of digital health in the Arab countries. Many gaps in research were found in Arab countries, which will be discussed subsequently. Digital health research was clustered around themes of big data and artificial intelligence; a lack of progress was seen in telemedicine and digital health. Furthermore, only a small proportion of these publications had principal or corresponding authors from Arab countries. A clear disparity in digital health research in the Arab world was evident after comparing these insights with our previous investigation on telemedicine research in the global context.

**Conclusion:** Telemedicine research is still in its infancy in the Middle Eastern countries. Recommendations include diversification of the research landscape and interdisciplinary collaborations in this area.

## Introduction

Provision of healthcare services to remote areas has been made a reality with effective coherence of the health profession and technology. Advancement of communication technology has given new momentum to the previously limited concept of “telemedicine.” In this golden era of information and communication technology, telemedicine is not only being used for diagnosing and treating ailments, but it has also led to the development of numerous health-related educational resources that aid in the advancement of medical education around the world, equipping professionals with the necessary knowledge and skills (1, 2). By the provision of uniform access to healthcare services in a variety of communities, a decrease in operational costs, support for healthcare providers, and a decrease in commute for healthcare have been seen. Over time, telemedicine technology has diversified, and innovation has led to the use of telemedicine in various specialties of medicine including teleradiology, teledermatology, telepsychiatry, and telepathology, among others. In fact, teleradiology, telemental health, distant patient monitoring, and teledermatology fields have shown greater success in telemedicine field than others (3). Despite the benefits of telemedicine, there exist wide variations in adoption of telehealth among different regions (4, 5). According to the World Health Organization, Europe leads the way on the number of functioning telehealth programs (4–6). Although there are dissimilarities, Eastern Mediterranean and Southeast Asian regions can benefit the most out of these telehealth facilities (6); there is a lack of research concerning the application of telemedicine in the Arab countries. There is a clear need to improve health outcomes and increase easier access to healthcare for all.

Arab countries share common culture and language and yet are exposed to wide discrepancies in the quality of life, education, and availability of healthcare amenities. At one end of the spectrum, there are oil-rich and natural resources–rich countries such as Saudi Arabia, Qatar, Kuwait, and the United Arab Emirates (UAE), whereas the other end includes countries such as Yemen, Palestine, and Syria, which are stricken by conflicts, famine, and poverty (7). This difference in the quality of life between different Arab nations is a gap that does not allow for an accessible healthcare system for all. Telemedicine in this region can be a key factor to achieve Sustainable Developmental Goal 3: “Ensure healthy lives and promote wellbeing for all, at all ages” (8). The rich member countries of Gulf Cooperation Council (GCC) have vast resources and have made large investments in health infrastructure consisting of medical schools, universities, and hospitals (9). In these countries, telemedicine has also been adapted in healthcare setups as a tool for the provision of health facilities to remote areas and bringing expertise from developed nations to aid in the management of health systems (10).

Quantitative measurement of research and literary contributions within a particular field can be used to gauge progression of that field providing vital public health knowledge and scope for application. Previous literature has reported that GCC countries have made substantial investments in healthcare and telemedicine (10). A previous systematic review found 306 studies on telemedicine published from the GCC region (10). Conversely, countries such as Yemen, Syria, Palestine, and Iraq fall behind GCC countries in health and technological infrastructure, making telemedicine an inaccessible necessity (11). No efforts have previously been made to gauge the progress and innovation in telemedicine in these countries. Therefore, the present study aims to analyze the scholarly work conducted in the Arab world, using reproducible statistical and scientometric analytical techniques.

This scientometric investigation aims to:

a) Identify important collaborative entities such as institutes, regions, and funders.
b) Identify major topics of research in this domain.
c) Identify important scholarly activity such as landmark research papers.
d) Provide recommendations and a future roadmap for digital health in Arab countries.

## Methods

### Scientometric Analysis

The field of “scientometrics” pertains to “quantitative study of science, communication in science, and science policy” (12). Using several reproducible techniques, it is possible to assess the influence of research articles, citations, and journals in a field (12). The information gathered can therefore be used to aid resource allocation and policy formation and allocate funds in areas where improvement is required (13). To the best of our knowledge, this is the first piece of work to analyze scientific research in the field of telemedicine across the Middle East.

### Operational Definitions

We considered scholarly articles pertaining to digital health divided into three domains: (a) telemedicine and telehealth, (b) artificial intelligence and machine learning, and (c) mobile health. For this review, we defined telemedicine and telehealth as per the Health Resources and Services Administration guidelines (14). Telemedicine was defined as remote clinical services, whereas telehealth referred to preventive, promotive, and curative care delivery including non-clinical applications of telehealth technology (14). Use of artificial intelligence and machine learning included, but was not limited to, clinical decision support systems and applications of big data. And mHealth or mobile health pertained to the use of smart or portable devices for health services and information such as use of health applications and software (15). Our research spans across these three major domains pertaining to the Middle East.

### Database Search

An electronic search of Web of Science (WoS, core database) was conducted by using an extensive search strategy (Table 1) comprising keywords specific to the Arab region, Arab countries, telehealth, medical conditions, and disorders, yielding a total of 1,630 search results, indexed through July 7, 2020. This extensive search strategy was designed to identify articles spanning the broad domain of digital health in the Arab countries. Our choice of database was limited to the core databases of WoS because it is a multidisciplinary database providing coverage to more than 21,349 journals, more than 78 million records, and more than 116,000 books and 220,000 conferences (16). This database is particularly important because in addition to detailed bibliographic records for every scholarly item, it also indexes their citations, allowing for cocitation analysis, which is central to our investigation (17).

**Table 1:** Search strategy for Web of Science (core database) search.

**Concept**	**Limiters**	**Keywords**
Region	CU	(Arab^*^ OR middle-east^*^ OR Algeria OR Bahrain OR Comoros OR Djibouti OR Egypt OR Iraq OR Jordan OR Kuwait OR Lebanon OR Libya OR Mauritania OR Morocco OR Oman OR Palestine OR Qatar OR “Saudi Arabia” OR Somalia OR Sudan OR Syria OR Tunisia OR “United Arab Emirates” OR Yemen)
Telehealth	TS	(Telemedicine OR “health informatics” OR “clinical decision support system” OR “health app^*^” OR “health software” OR tele-health OR telerehabilitation OR telecommunication OR “text messag^*^” OR “wireless” OR “machine learn^*^” OR “artificial intelligence” OR “remote consultation” OR “mobile health” OR mHealth OR eHealth)
Disorders	AB/TI/TS	(chronic OR “chronic disease” OR illness^*^ OR “chronic disease” OR respiratory OR pulmonary OR kidney OR cerebrovascular OR infectio^*^ OR cancer^*^ OR metabolic OR gastr^*^ OR cardiac OR hypertens^*^ OR asthma^*^ OR COPD OR neuro^*^ OR coronary OR diabetes OR urinary OR urolog^*^ OR reproductive OR cardiovascular OR skin OR dermatolog^*^ OR psychiatr^*^ OR mental OR joint OR hormon^*^ OR “heart disease” OR disease^*^ OR endocrin^*^ OR neoplas^*^ OR communicable OR non-communicable OR psych^*^ OR patholog^*^ OR biochem^*^ OR disorder^*^ OR gyneco^*^ OR obstetrics OR neuro^*^ OR ophthalmolo^*^ OR neuro^*^ OR eye^*^ OR ear^*^ OR nose OR throat OR hematolog^*^)

### Analysis

CiteSpace (5.7.R1, Drexel University, Pennsylvania, USA) is a Java-based application, a user-friendly tool for conducting scientometric analyses. It has recently gained popularity for its use in visualization of bibliographic data and knowledge mapping by leveraging the theory of cocitation analysis (17, 18). According to the theory of cocitation analysis, two documents are related to each other if they are cited together in another document (18). To visualize bibliographic data of digital health in Arab countries, bibliographic records were obtained from the WoS and were fed into CiteSpace. Using time slicing, the bibliographic data were analyzed from January 2000 to July 2020, with each time slice represented by top 50 cited articles per year. For the processing of data, term sources were extracted from the title, abstracts, and author keywords for each article. To gain an insight into top institutions, countries, and keywords for published research, respective node types were selected. In resulting visualizations, articles were presented as nodes and links as edges. These visualizations provide several insights to identify pattern and trends of research in a domain. Purple rings correspond to new theories and concepts in a field, centrality bursts present hot topics of research with increased research activity in a small period, and centrality rings with centrality values >0.1 relate to influential studies in their collaborative networks (18). In addition, each node is presented with a tree ring providing details on yearly pattern of citations, with the size of the ring corresponding with number of citations (18).

Moreover, to gain insights into influential research works within this domain, separate analyses were run, where node type was indicated as reference. Network analyses were run with the link reduction method using pathfinder scaling; where link strength was calculated using *Cosine* similarity measure to measure document similarity in text analysis (18). Cluster analysis was then run to identify clusters of research articles. Clusters of research produced in these analyses were considered parsimonious based on their silhouette index, modularity, and number of articles (18).

## Results

**Figure 1:**
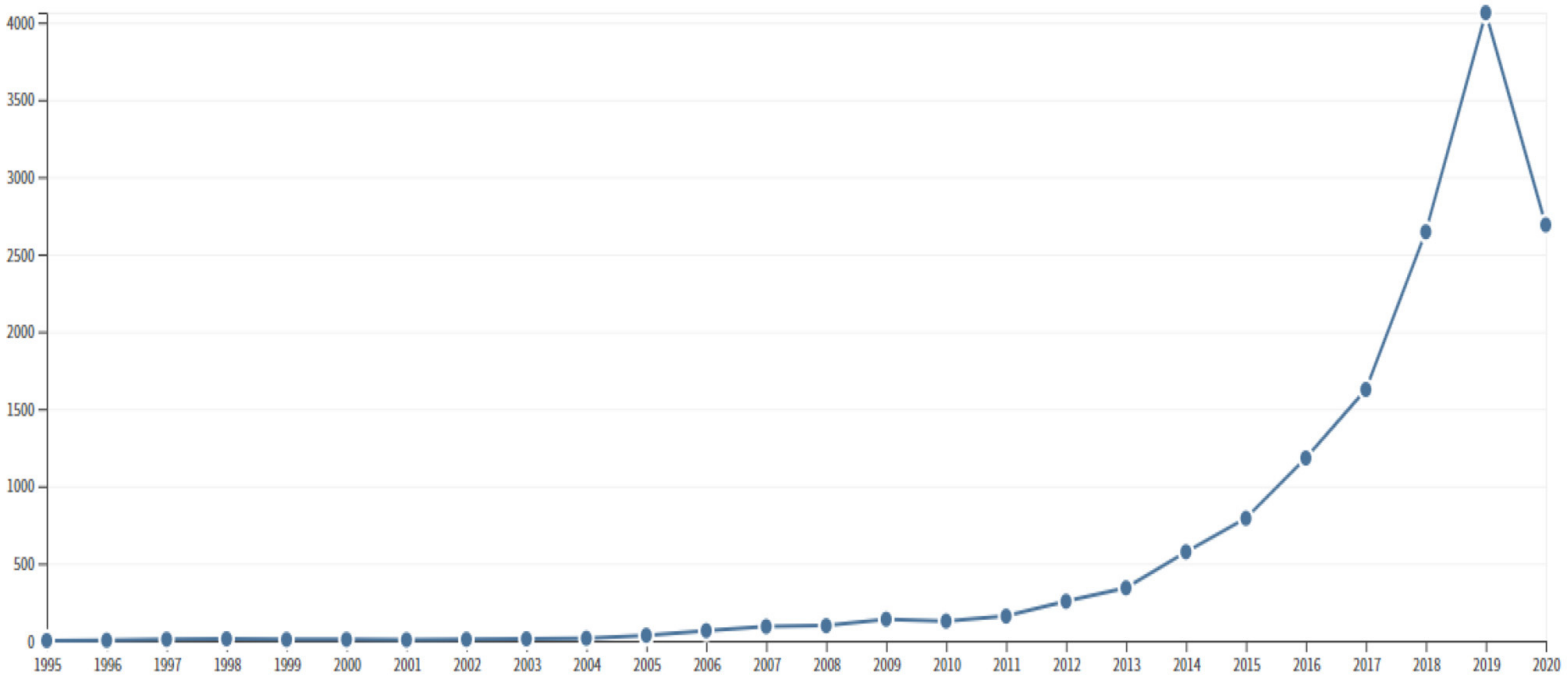
Citation pattern for 1,630 publications on telemedicine in Arab countries through July 7, 2020.

**Figure 2:**
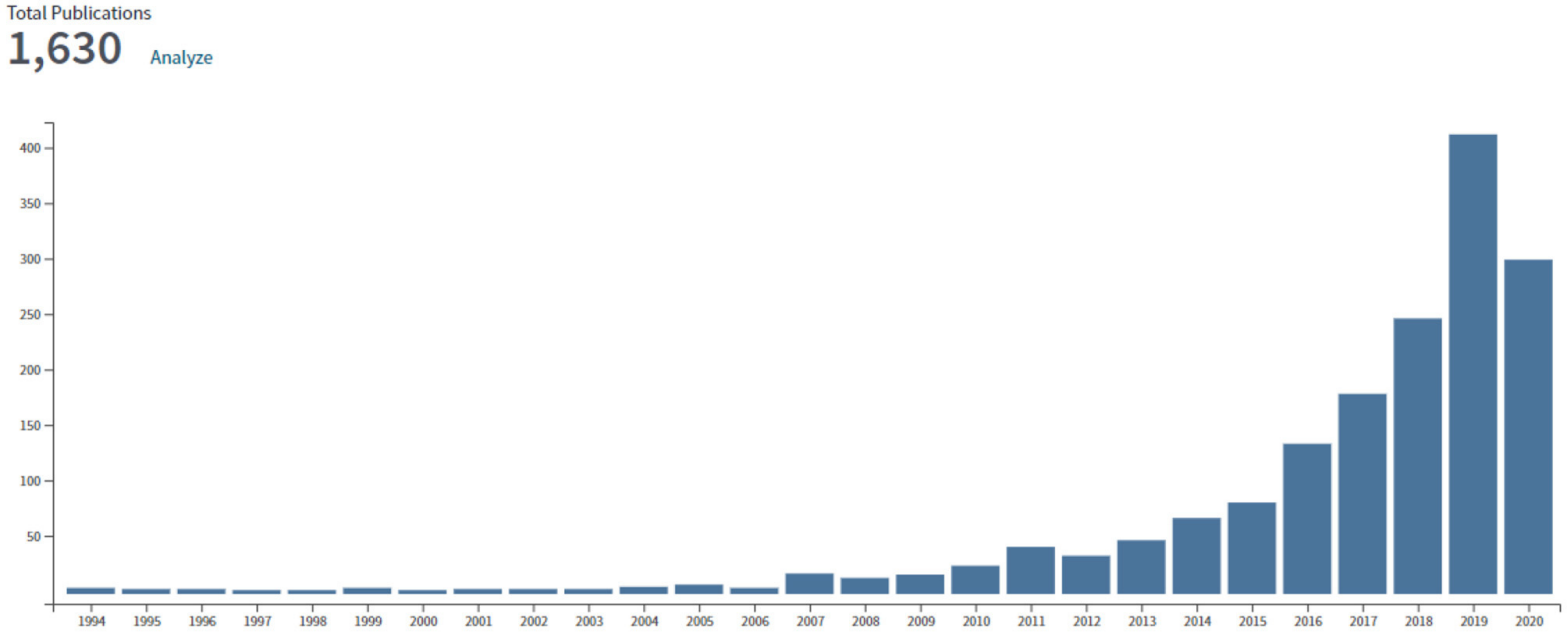
Publication pattern for 1,630 publications on telemedicine in Arab countries through July 7, 2020.

The publications pertaining to digital health in the Arab world were cited for a total of 14,967 times, yielding an h-index of 48 and an average citation per item of 9. An analysis of publication and citation patterns (Figures 1, 2) revealed a yearly increasing trend in research activity on telemedicine, after the year 2011. Both the publication and citation activity showed a sharp rise after the year 2015.

### Regional Trends in Publications

An analysis of country-wise trends in publication revealed important insights on research activity based on telemedicine. In terms of publication activity, the Kingdom of Saudi Arabia (KSA) led the region with 541 citations followed by Egypt (*n* = 318), UAE (*n* = 167), and Qatar (*n* = 130). Iraq, Tunisia, Jordan, Algeria, Lebanon, and Morocco accrued a count between 50 and 100 publications. None of the countries from the Arab region were found to be central in their network.

**Figure 3:**
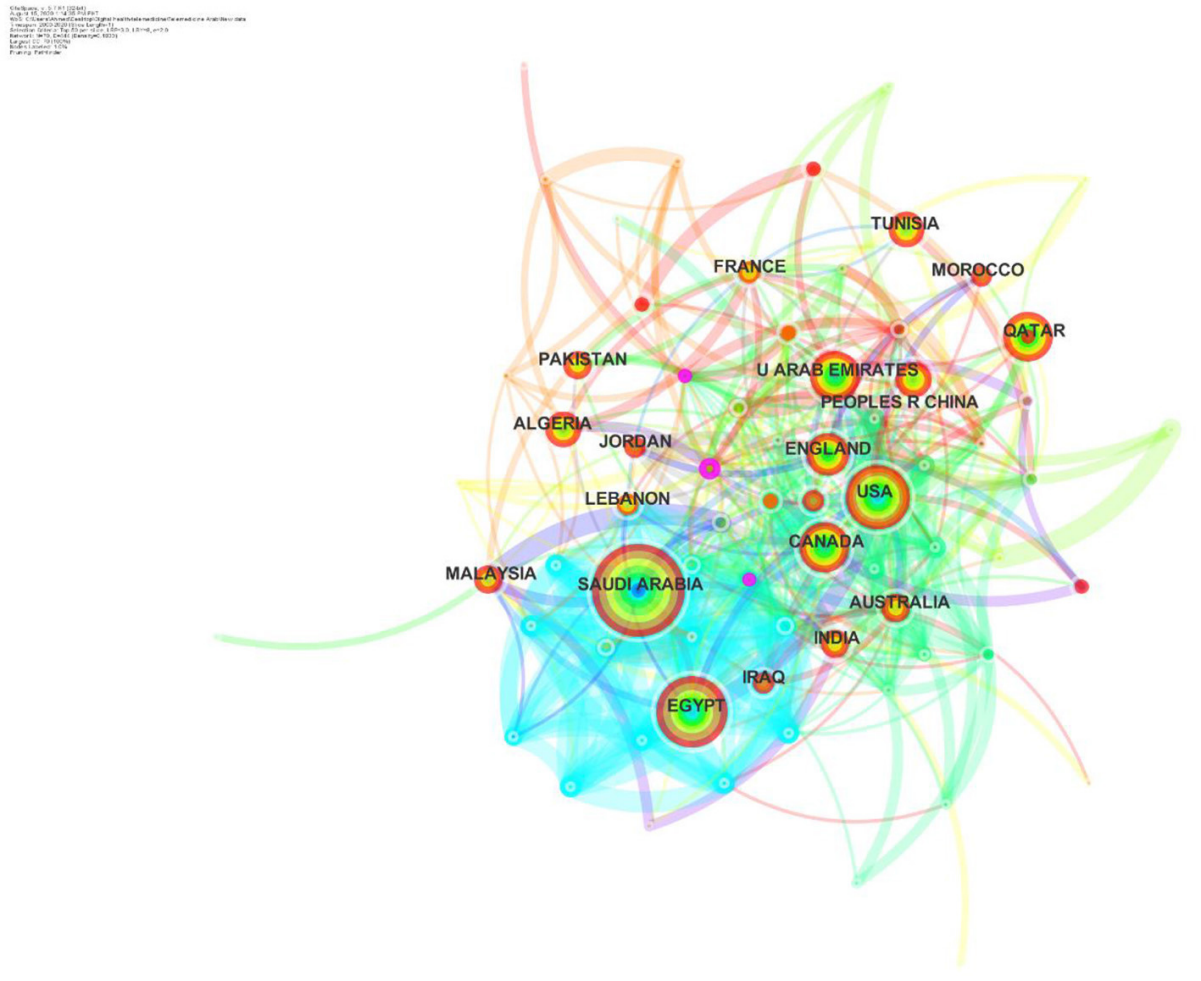
Central countries in the area of digital health in the Arab world.

An in-depth analysis of collaborative patterns among countries revealed frequent collaborations with countries in other continents. Saudi Arabia, for instance, revealed collaborative links with countries such as USA, Canada, UK, China, Malaysia, New Zealand, and Pakistan. The collaboration of Arab countries in research, as a collective, led to six countries yielding high centrality values (>0.10): Singapore, Germany, Japan, Malaysia, Brazil, and South Africa (Figure 3). None of the countries in the Arab region scored a centrality value >0.10. Nine countries revealed citation burst activity on telemedicine in Arab countries: USA, Jordan, Canada, Algeria, and France. Their citation activity lasted between 5 and 10 years, whereas citation burst activity of Egypt, Lebanon, Kuwait, and Qatar ranged between 1 and 5 years (Table 2).

**Table 2:** Countries showing citation bursts.

**Countries**	**Year**	**Strength**	**Begin**	**End**	**2000–2020**
USA	2000	6.826	2005	2013	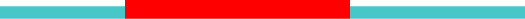
Egypt	2000	3.7234	2005	2006	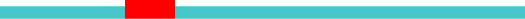
Jordan	2000	7.5285	2007	2012	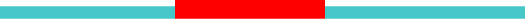
Canada	2000	5.8829	2007	2011	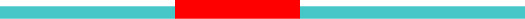
Algeria	2000	5.1469	2007	2014	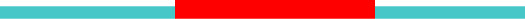
France	2000	5.3921	2008	2014	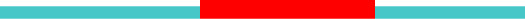
Lebanon	2000	5.4265	2010	2013	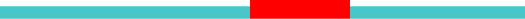
Kuwait	2000	4.3153	2011	2014	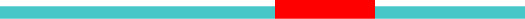
Qatar	2000	4.8755	2013	2016	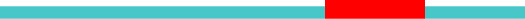

### Institutional Trends in Telemedicine Research

**Figure 4:**
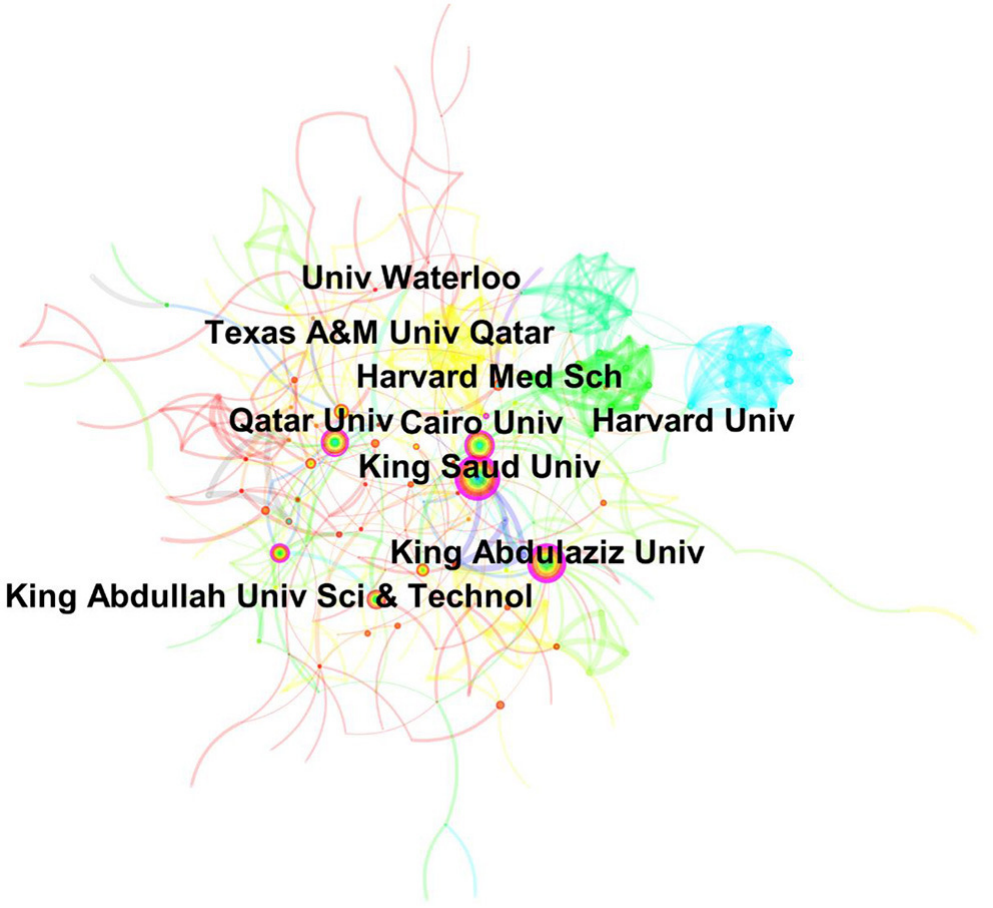
Central universities in the area of digital health in the Arab world.

Among institutions, the most central institutions contributing to telemedicine in the Arab world were King Abdul-Aziz University, King Saud University, and King Abdullah University of Science and Technology from KSA, Cairo University in Egypt, and Qatar University in Qatar. Foreign central institutes included University of Waterloo in Canada and Harvard Medical School in USA (Figure 4).

Most frequently publishing institutes included King Saud University (*n* = 152), followed by Cairo University (*n* = 90), King Abdul-Aziz University (*n* = 86), Qatar University (64), KAUST (*n* = 61), King Fahd University of Petroleum (*n* = 48), Khalifa University of Science and Technology (*n* = 37), American University of Beirut (*n* = 33), and Mansoura University (*n* = 38).

### Funders

Only two funding bodies were found to be central (>0.10) to telemedicine research in Arab countries: (a) National Natural Science Foundation of China and (b) Deanship of Scientific Research at King Saud University. On the basis of frequency of funding awards, top funding body included Deanship of Scientific Research at King Saud University (*n* = 63), National Natural Science Foundation China (*n* = 40), US Department of Human Health Services (*n* = 35), National Institutes of Health USA (*n* = 34), National Science Foundation (*n* = 24), European Union (*n* = 21), Natural Sciences and Engineering Research Council of Canada (*n* = 19), and King Abdullah University of Science and Technology (*n* = 16).

### Keyword Analysis

A total of 12 keywords were found to be central to the domain of telemedicine research in the Arab world, accruing a centrality score >0.10. These 12 keywords were categorized into the following:

Topic: telemedicine, system, disease

Machine learning: machine learning, design, algorithm, prediction, performance, model, knowledge

Communication: communication, wireless sensor network

An analysis of top cited keywords (>30) revealed the following concepts in the domain of machine learning: classification, system, model, prediction, neural network, algorithm, network, optimization, artificial intelligence, performance, artificial neural network, feature selection, deep learning, selection, support vector machine, health, management, design, big data. As per citation burst analysis, frequent keyword use in telemedicine pertained to machine learning, wireless networks, and most recently big data and data mining (Table 3).

**Table 3:** Citation burst of keywords.

**Keywords**	**Year**	**Strength**	**Begin**	**End**	**2000–2020**
Neural network	2000	3.2711	2008	2013	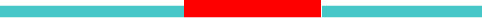
Wireless	2000	7.6545	2009	2016	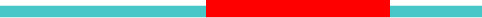
Knowledge	2000	3.9277	2011	2015	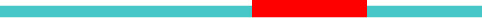
Power control	2000	4.3244	2011	2016	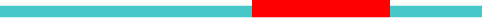
Identification	2000	3.3572	2012	2013	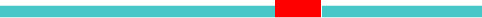
*Ad hoc* network	2000	3.7355	2012	2014	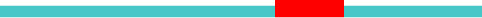
Diversity	2000	3.8731	2013	2016	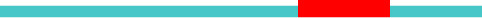
Design	2000	5.0496	2014	2015	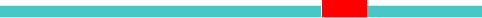
Channel	2000	5.8542	2014	2016	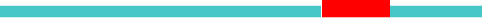
Adherence	2000	3.1567	2015	2017	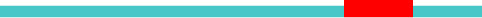
Resource allocation	2000	4.782	2015	2016	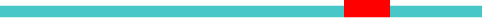
Management	2000	3.1578	2015	2018	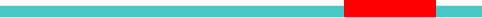
Association	2000	3.7947	2015	2018	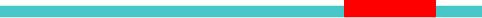
Data mining	2000	3.2313	2017	2020	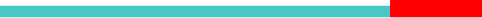
Brain	2000	3.0928	2018	2020	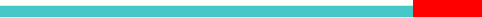

### Clusters of Research in Telehealth

Only one major cluster comprising four subclusters was identified in the domain of telehealth research in the Arab countries. All these clusters represented the broad category of machine learning and were found to be parsimonious with a silhouette index >0.90. These subclusters were termed as convolutional neural networks (clusters 0 and 2), brain tumor detection (cluster 2), and feature selection (cluster 8).

**Figure 5:**
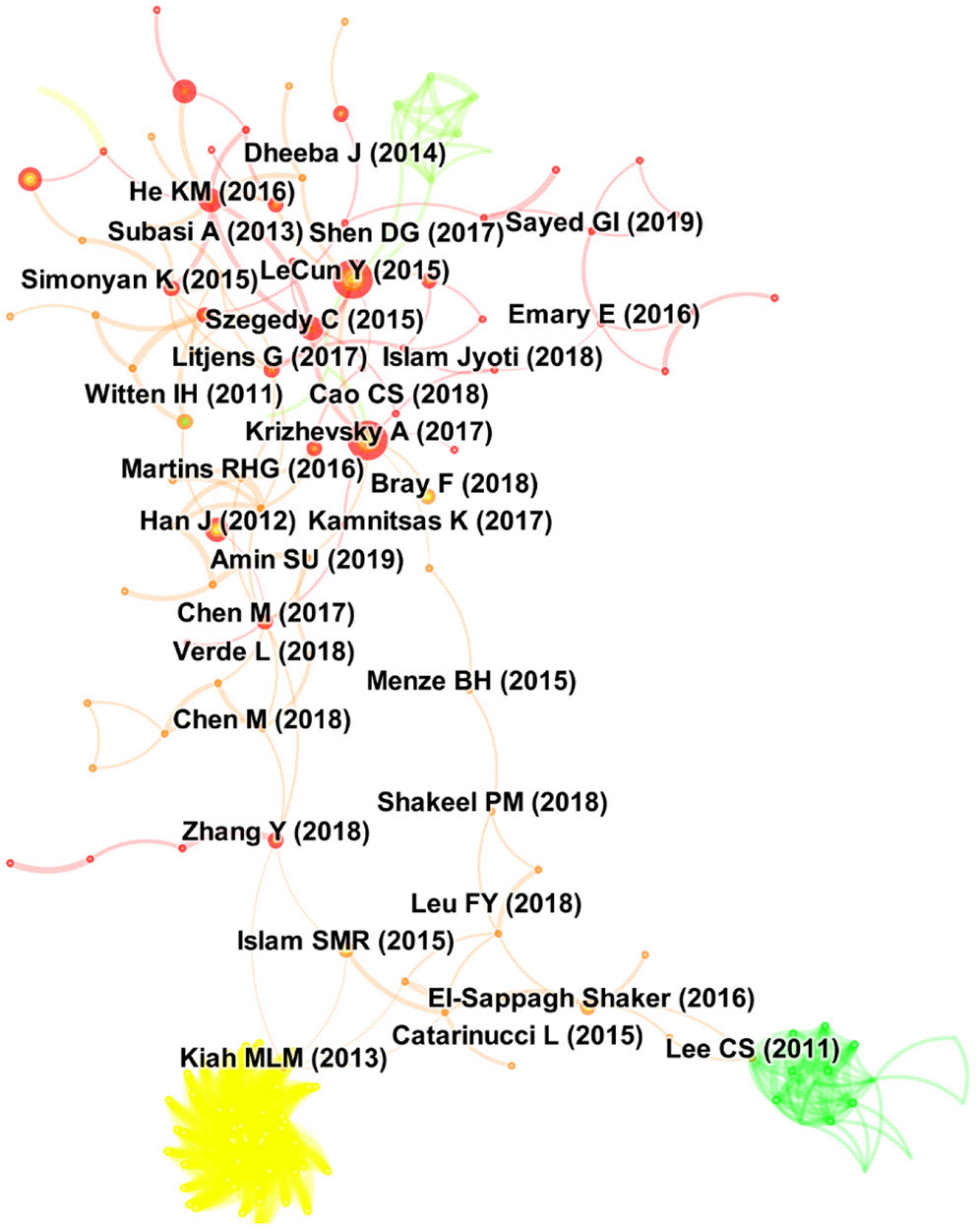
Central publications in the area of digital health in the Arab world.

**Figure 6:**
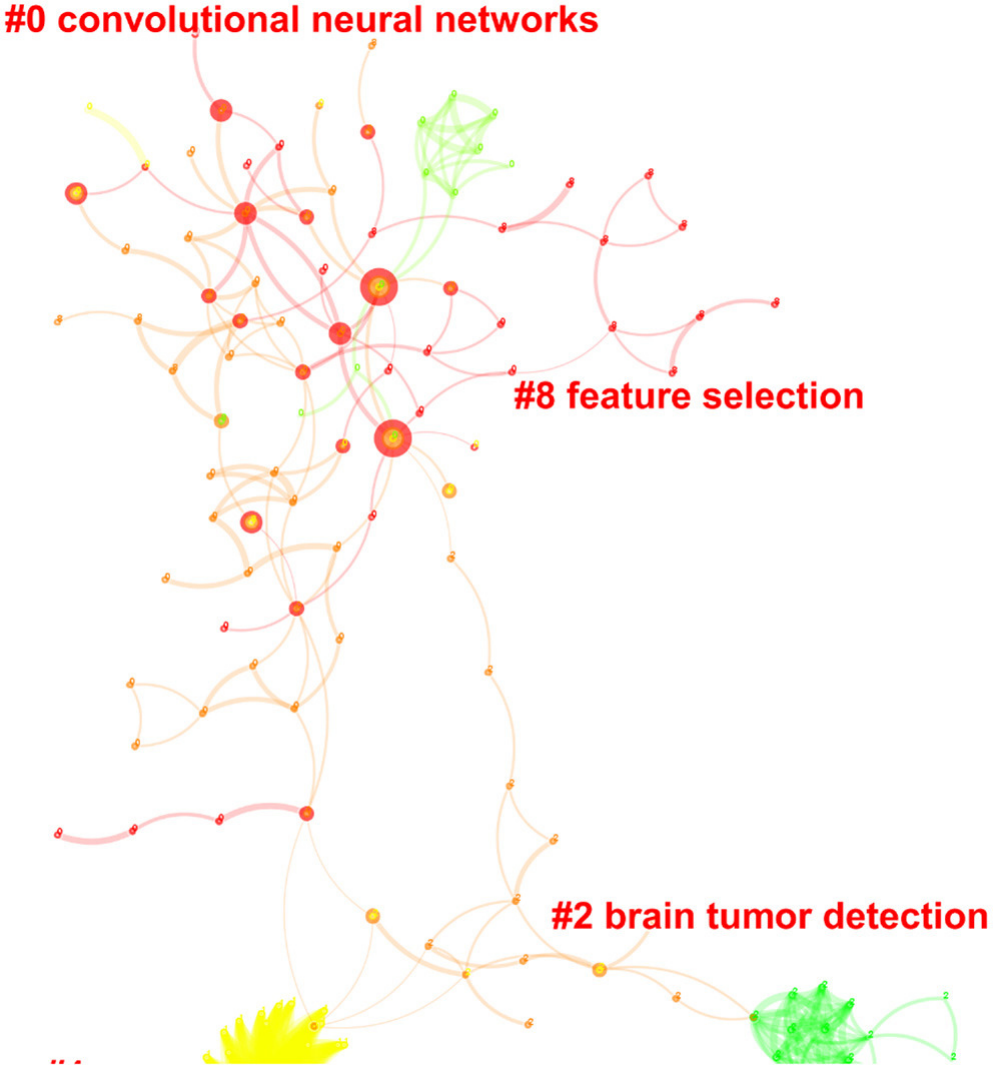
Clusters of research in the area of digital health in the Arab world.

Top citer for the first cluster was (19) from Helwan University, Cairo, Egypt: presenting a review on swarm and evolutionary computing approaches for deep learning (19). In cluster 2, the most active citer was El-Sappagh et al. based at Minia University in Egypt, who published three review papers on medical case reasoning frameworks, SNOMED CT ontology and mobile health technologies for diabetes mellitus (20–22). The last subcluster was actively cited by Syaed et al. based at Cairo University in Egypt, who published his empirical work on binary whale optimization algorithm and binary moth flame optimization with clustering algorithms for clinical breast cancer diagnoses (23) (Figures 5 and 6).

### Lessons Learned From Central Publications

None of the publications in this collaborative domain of research yielded optimal centrality levels (>0.1) (Figure 5). In addition, no hotspots of research or purple nodes were identified. Therefore, we sought to explore publications yielding centrality values >0.01. There were a total of 25 publications with centrality values >0.01. It was interesting to note that only a small proportion of these publications had principle or corresponding authors affiliated with Arab institutions. Most of these publications pertained to the use of big data and machine learning techniques in healthcare. A total of 18 studies pertained to the use of machine learning by applying techniques such as deep learning specifically neural networks. Four studies focused on data mining, anonymization, and security measures for electronic medical records (*n* = 2) and wireless sensors (*n* = 1). Among studies focusing on disease detection and screening, a variety of techniques and topics were explored. Chen et al. using hospital-based data, proposed a convolutional neural network–based multimodal disease risk prediction algorithm, with an accuracy of 94.8%. Diabetes mellitus diagnosis ontology development was undertaken by El-Sappagh et al. for quick and accurate diagnosis of diabetes mellitus. Lee et al. proposed a novel fuzzy expert system for diabetes decision support application. Subasi proposed a hybrid machine learning method for improved classification of EMG signals to diagnose neuromuscular disorders. Central nervous system pathologies were explored in three publications focusing on Alzheimer disease, detection of brain tumors using magnetic resonance imaging (MRI) scans and brain disease segmentations. Two books edited by Han and Witten and published by the MOR KAUF D, entitled “Data Mining: Concepts and Techniques” and “Data Mining 4th Edition Practical Machine Learning Tools and Techniques,” were found to be central and most frequently cocited by Arab authors. Detailed objectives and lessons learned from these studies are presented in Supplementary Table 1.

## Discussion

### Summary

This scientometric investigation is one of the first works that analyze the scholarly contributions in the field of digital health in the Arab world. The present analyses found a lack of innovation in the field of digital health in Arab countries including a wide range of gaps in research, which are discussed subsequently. Digital health research was clustered around themes of big data and artificial intelligence, whereas a lack of innovation in telemedicine and mHealth was observed. It was also interesting to note that only a small proportion of these publications had principal or corresponding authors from the Arab countries.

### Top Players in Digital Health Research

Institutions based in KSA led the region in digital health research followed by Egypt, UAE, and Qatar, which were nonetheless supported by other countries, particularly the USA, UK, Canada, China, and Malaysia. These strong collaboration links with the Western world, as well as Southeastern countries of China and Malaysia, point to the knowledge exchange hubs for the Arab world in this field. Having a strong economy and willingness to finance digital health in their countries are two important factors for the high research output in these countries. Besides these factors, a strong vision and defined roadmaps adopted by these countries may have accelerated digital health research.

These defined roadmaps led to launch of several projects and national-level policies in these countries; for instance, in Qatar, the field of digital health gained national attention after 2010, specifically from 2011 to 2013, with the launch of “Project 2.4. E-Health Establishment” as part of the National Health Strategy 2011–2016 with the goals of creating an effective, integrated national Health Information Exchange system that enables participation of all healthcare providers in Qatar and ensures national alignment for implementation. Anticipated outcomes include national e-health regulations, education, and development of e-health technical architecture and a national health data warehouse (24). In Saudi Arabia, a national 10-year e-health strategy was launched in 2011 to prioritize clinical automation, data centers, and a nationwide electronic health records system. In a similar vein, the health authorities in the UAE, despite the absence of a national health plan, are working toward computerization of healthcare facilities and data sharing (10).

### Comparison With Global Literature

In consonance with the global scientometric investigations on digital health (25), one of the top clusters of research relates to breast cancer. Our analyses also identified landmark publications in the domain of deep learning using MRI, an essential component of the field of teleradiology. Similarly, clinical decision support systems particularly for diabetes, as well as development of wireless sensors, also achieved centrality in our analyses. These results correspond with topics of global importance as reported by Fang, in their publication on evolution of digital medicine in the world (25).

Our results are also in consonance with Weber et al. (10), who reported e-Health research trends in the six countries belonging to the GCC. Some heterogeneity in the present results and in those of Weber et al. exists mainly owing to the restriction of Weber and colleagues' investigation to oil-rich and stable economies in the Arab region. Weber et al. employed qualitative investigation, with manual coding of data, and found the significant themes to be benefits of e-health, implementation, and satisfaction with electronic health records, technology-delivered medical education, and information security (10). They also reported that most of the studies were either overviews, case studies, or descriptive articles, which were not very frequently cited in literature and therefore failed to gain centrality in our analyses.

A disparity in digital health research in the Arab world was evident after comparing these insights with our previous investigation on telemedicine research in the global context (26). The telemedicine-related bibliographic data when visualized revealed important insights with research in teleradiology and telepathology as important clusters pre-2010, whereas telepsychiatry, teledermatology, and deep learning gained momentum from 2010 to 2020. Such variation in digital health research is not seen in the Arab world, which is restricted to simpler study designs, review papers, and cross-sectional studies.

### Strengths of Our Work

The importance of our work lies in demonstrating that telemedicine research is still in its infancy in the Middle Eastern countries. This work lays out the need for a strong defined vision for progression in countries where telemedicine requires more research. Importantly, these countries should focus on diversification of the research landscape and foster interdisciplinary collaborations in this area, to allow for the growth of telemedicine. Moreover, the lack of a diverse, pool central research funding bodies may deny many researchers the ability to fund research that otherwise would diversify the field of telemedicine and telehealth within the Arab countries. The present investigation has enabled a clear insight into which Middle Eastern institutions play the greatest role in telemedicine and the importance of a collaborative web for institutes and funders within this region. Because of its infancy, researchers can endeavor to mold their research styles to advance collaboration and improve telemedicine.

### Gaps in Research and Way Forward

This scientometric investigation aimed to map the current trends in digital health research in Arab countries using reproducible statistical methods. This investigation is important as it plays a key role in the mapping of scholarly activity in Arab countries. This is very relevant to identify current state of research, areas where more funding, infrastructure, and capacity building are required. All of which play an integral role in advancing scientific literature and therefore having an impact on the practice of healthcare. Mapping the scholarly work performed in this area is integral to be able to identify areas for further research to be performed. All in all, we identified a paucity in the quality of work conducted in digital health in Arab countries.

Based on our analysis, no important/central piece of work ever published from any Arab institute was found.

**Figure 7:**
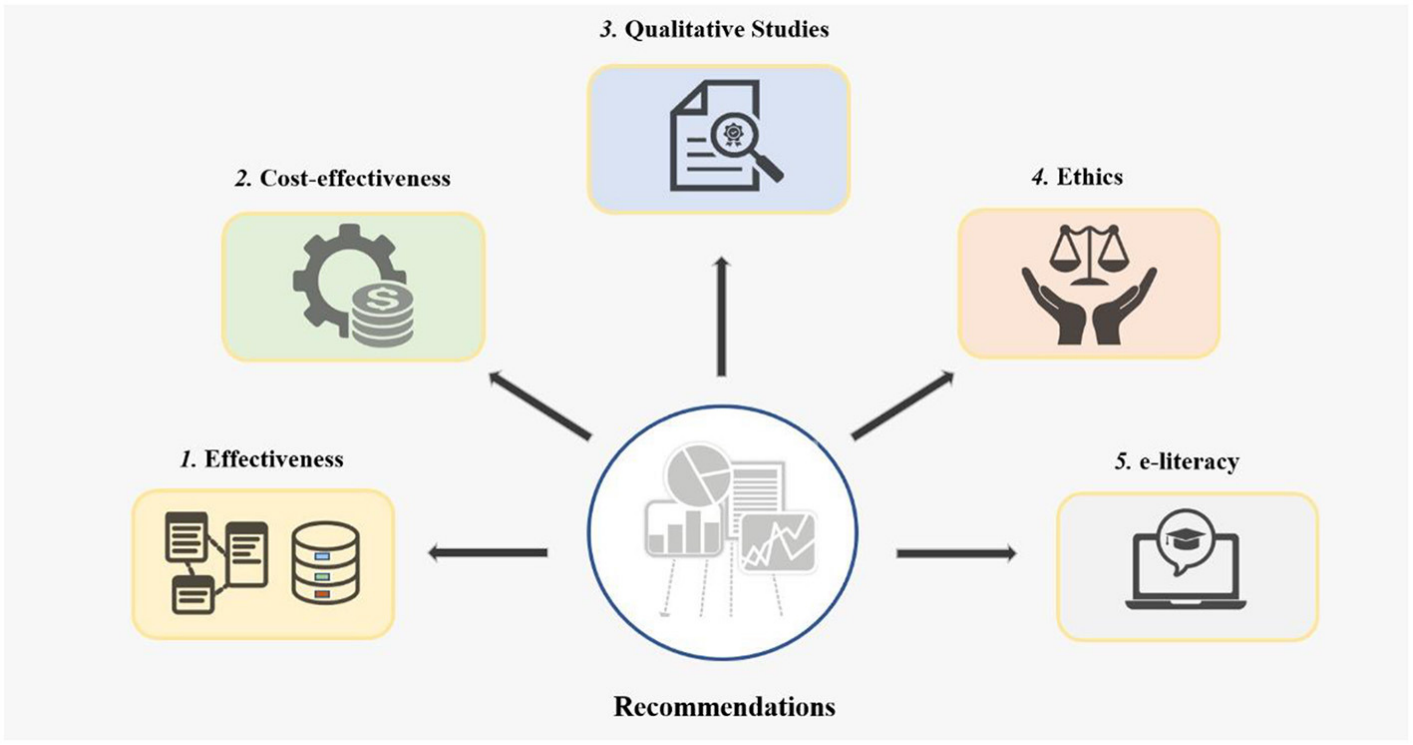
Recommendations to bridge the gap.

We identified important research gaps in digital health research based on our scientometric analyses (Figure 7), also corroborated by previous research (10, 26):

a) Trial data: We found a lack of controlled trials of medical devices, algorithms, and telemedicine technology in hospital centers in the Arab world. If we are to estimate precise effect sizes for these technologies, it is important to develop this field of research in these countries.
b) Cost-effectiveness: Cost-effectiveness studies are needed to gauge cost-effectiveness of digital health programs in real-world settings. This is particularly important when discussing telemedicine infrastructure in relatively weaker economies in the Arab world.
c) Qualitative studies: These studies are required to explore the nuances associated with acceptability, uptake, and feasibility of digital health programs in the Arab world. It is important to understand the end-consumer level preferences to ensure these programs are acceptable for uptake and scale-up in this region.
d) Ethics: Gender and religious issues are important considerations in context of the Muslim majority Arab countries. To ensure access of the female population to the digital health infrastructure, it is important that the format of delivery is sensitive to the cultural and religious context of the setting. In addition, concerns related to information security, confidentiality, and privacy are other important arenas in this domain.
e) E-literacy: Drives to improve e-health literacy and readiness to learn and adopt information technology for both the doctors and general public are crucial for success of digital health programs. Such drives should also be made a part of undergraduate and post-graduate curriculum to ensure physician competency in these domains.
f) Policy-making: The Arab world should develop policies and roadmaps for the implementation of digital health programs. Lack of such policies and legal frameworks lead to innovations that are not integrated into existing health systems, leading to poor uptake of telemedicine programs.
g) Collaboration: We recommend increasing collaboration between Arab countries that share important cultural similarities and not only rely on Western and Southeastern countries.

## Data Availability Statement

The original contributions generated in the study are included in the article/Supplementary Material, further inquiries can be directed to the corresponding author.

## Author Contributions

NA and AW conceived the idea. NA collected data and supervised the analyses. AW performed the analyses and interpreted results. SM, AJ, BP, and SK wrote the initial draft of the manuscript. JC and NA critically reviewed and edited the manuscript. All authors approved the final manuscript for submission.

## Conflict of Interest

The authors declare that the research was conducted in the absence of any commercial or financial relationships that could be construed as a potential conflict of interest.
