# Supplementary material for: Telemedicine in Arab Countries: Innovation, Research Trends, and Way Forward
Source: Front Digit Health. 2021 Jan 25;2:610837. doi: 10.3389/fdgth.2020.610837 (PMC8521801; doi:10.3389/fdgth.2020.610837)
Supplement: Supplementary file 1 [file Table_1.docx]

Supplementary Table 1: Lessons learnt from top publications

| **Centrality** | **Author** | **Source** | **Theme** | **Lesson learnt** |
| --- | --- | --- | --- | --- |
| 0.05 | Kiah MLM | J Med Syst | Security solutions for EMRs | Hybrid technique for security of EMR provided enhanced security, integration and accessibility |
| 0.04 | Chen M | Ieee Access | Machine learning algorithms for predicting disease outbreaks | Diversity of hospital data affects the accuracy of disease risk prediction such as better features description of the disease improves the accuracy of disease prediction. Convolutional neural network based multimodal disease risk prediction (CNN-MDRP) algorithm using structured and unstructured data from hospital when compared to several typical prediction algorithms showed prediction accuracy of this proposed algorithm to be 94.8% |
| 0.04 | Zhang Y | J Netw Comput Appl | Authentication for Smart healthcare systems | To improve the accuracy and efficiency of ECG-based authentication, this paper proposed a parallel approach that incorporates multiple features for smart healthcare systems. |
| 0.02 | Litjens G | Med Image Anal | Deep learning algorithms for analysis of medical images | End to end learning convulutional neural networks (CNNs) have become the preferred approach for medical imaging interpretation. Such CNNs are often integrated into existing image analysis pipelines and replace traditional handcrafted machine learning methods. |
| 0.02 | El-Sappagh Shaker | Appl Inf | Diagnosis ontology development | An attempt to make knowledge based for quick and accurate diagnosis of diabetes. The ontology is comprehensive, as it contains all diabetes-related complications, laboratory tests, symptoms, physical exams, demographics, and diagnoses. DDO (Diabetes Mellitus Diagnosis Ontology) is the first reported diabetes disease ontology developed to represent different disease aspects in a formal logical format. |
| 0.02 | Catarinucci L | Ieee Internet Things | Automatic monitoring healthcare system development | Smart Hospital System (SHS) architecture for automatic monitoring and tracking of patients, personnel, and biomedical devices within hospitals and nursing institutes has been proposed. The achieved results demonstrate the appropriateness of the proposed system to perform not only identification and tracking of patients, nursing staff, and biomedical devices within hospitals and nursing institutes, but also to provide power-effective remote patient monitoring and immediate handling of emergencies. |
| 0.02 | Lee CS | Ieee T Syst Man Cy B | Diagnosis ontology development | The proposed fuzzy ontology method can analyze data and further transfer the acquired information into the knowledge to simulate the thinking process of humans. The results further demonstrate that the proposed method works more effectively for diabetes application than previously developed ones. |
| 0.01 | Krizhevsky A | Commun Acm | Convulated Neural Networks development | Deep CNN is capable of achieving record-breaking results on a highly challenging dataset using purely supervised learning to classify object images. |
| 0.01 | LeCun Y | Nature | Machine learning | Deep learning of computational models have dramatically improved the state-of-the-art in speech recognition, visual object recognition, object detection and many other domains such as drug discovery and genomics |
| 0.01 | He KM | Proc Cvpr Ieee | Training of neural networks | Reformulating the layers of deep neural networks as learning residual functions with reference to the layer inputs, instead of learning unreferenced functions. It provided comprehensive empirical evidence showing that these residual networks are easier to optimize, and can gain accuracy from considerably increased depth. |
| 0.01 | Szegedy C | Proc Cvpr Ieee | Neural Network Architecture | Convulational deep neural network named as 'Inception' achieves the new state of the art for classification and detection in the ImageNet Large-Scale Visual Recognition Challenge 2014 (ILSVRC14). This architecture improves usage of the computing resources inside the network. This design increased depth and width of computing network with same computational budget. |
| 0.01 | Han J | Mor Kauf D | Data mining | This book describes concepts and methods pertaining to data mining. |
| 0.01 | Witten IH | Mor Kauf D | Data mining | This book describes concepts and methods pertaining to data mining. |
| 0.01 | Subasi A | Comput Biol Med | Machine learning application in biomedical | Proposed Support Vector Machine (SVM) model when compared to conventional learning methods showed superior results of accuracy in terms of EMG use for diagnosing neuromuscular diseases. |
| 0.01 | Emary E | Neurocomputing | Feature selection | Comparison of two versions of Binary Gray Wolf optimizers (bGWO1 and bGWO2) to popular methods such as particle swarm optimization (PSO) and genetic algorithms (GA) showed that binary versions outperformed PSA & GA in search capability. Binary Gray Wolf optimizers' feature selection method shows superior separability. |
| 0.01 | Leu FY | Comput Electr Eng | Wireless Body Sensor Networks (WBSNs) for healthcare monitoring, prevention and management | Sensor system named as Mobile Physiological Sensor System (MoPSS) is developed for real time patient monitoring. MoPSS provides better solution for health management. This monitoring system helped understand the value of wearable sensors for provision of real time physiological data to help patients timely and efficiently. |
| 0.01 | Islam Jyoti | Brain Inform | Imaging analysis by deep convolutional neural networks | The proposed model od Brain MRI analysis can identify various stages of Alzheimer's disease. This outperformed various basline models as it showed immproved performance for multi-class classification. This can be applied to classification of other medical problems. |
| 0.01 | Menze BH | IEEE T Med Imaging | Brain tumours imaging analysis | There was considerable disagrement between different human evaluators in classification of variety of tumor subregions. Different sub region tumors were analyzed better using different algorithms and there was no single algorithm proved to be better than for all subregions unanimously |
| 0.01 | Verde L | IEEE Access | Machine learning and disease detection | Detection and evaluation of comparison of variety of machine learning techniques useful for detection of pathology of voice. Support Vector Machine algorithm or the Decision Tree algorithm showed best accuracy for detection of voice diseases |
| 0.01 | Bray F | Ca-Cancer J Clin | Global disease burden estimate | Cancer is a significant cause of morbidity and mortality worldwide. Estimation of the new cancer cases and cancer deaths was predicted for year 2018. For both genders, lung cancer is the most diagnosed type of cancer & also the leading cause of cancer mortality. In males, as per incidence, lungs cancer is the most frequent type and then comes prostate & colorectal cancer. For female gender, breast cancer shows the highest incidence and cause of cancer mortality. For males, liver and stomach cancers showed the highest mortality. Economic development & lifestyle are the factors leading to variation in frequency of type of cancer diagnosed and cause of cancer death among different countries and different regions of the same country. |
| 0.01 | Martins RHG | J Voice | Disease aetiology and diagnosis | Different factors associated with voice disorders were studied like age, gender, profession, smoking prevalence etc. Gender based incidence of various voice disorders was also calculated. |
| 0.01 | Shakeel PM | J Med Syst | Security and privacy of Health record | Internet of things (IoT) based security network Learning based Deep-Q-Network (LDQN) utilized to improve security of health related devices. It helped separate malware activities from secured data and thus improved efficiency of IoT-health data system. LDQN lead to increased detection of malware. |
| 0.01 | Kamnitsas K | Med Image Anal | Machine learning for disease detection | This study proposes a convolutional network analysis based model for accurate brain lesion segmentation |
| 0.01 | Amin SU | IEEE Access | Convoluted Neural Networks development | Acquisition of multimodal medical data by use of senors which informs about patients' condition that helps deciding patients' needs. This system informs all concerned individuals about patient condition so that appropriate measures can be implemented |
| 0.01 | Cao CS | Genom Proteom Bioinf | Deep learning application in biomedicine | Deep learning based convolutional neural networks are being used in biomedical image analysis due to their ability of spatial information analysis. Further application of deep learning concepts can improve disease prediction, prevention, diagnosis and management. It can also aid in predicting epidemics. |
